# Solitary hepatic lymphangioma in an 8-month-old child

**DOI:** 10.11604/pamj.2015.20.440.6111

**Published:** 2015-04-30

**Authors:** Mohamed Zouari, Mahdi Ben Dhaou

**Affiliations:** 1 Department of Pediatric Surgery, Hedi Chaker Hospital, 3029 Sfax, Tunisia

**Keywords:** Cystic lymphangioma, liver, children

## Image in medicine

The cystic lymphangioma of the liver is an extremely rare benign tumor that usually belongs to a systemic lymphangiomatosis. An 8-month-old boy came to our Department due to abdominal distension. On physical examination, a palpable abdominal mass was noticed on the right upper quadrant, with voluntary guarding and no peritoneal irritation. Laboratory tests did not produce any positive findings. Abdominal ultrasound revealed a hepatic cyst measuring 14.9 cm × 10.7 cm × 9.7 cm in the right hepatic lobe. An enhanced abdominal CT scan confirmed a cystic hepatic lesion with no capsule. There were no definite septations or solid elements within the cyst. At laparotomy, a giant unilocular cyst arising from the visceral surface of the right lobe of liver extending into whole abdominal cavity and pelvis was observed. Complete excision of the cyst was performed. The pathology report defined the tumor as a cystic hepatic lymphangioma.

**Figure 1:**
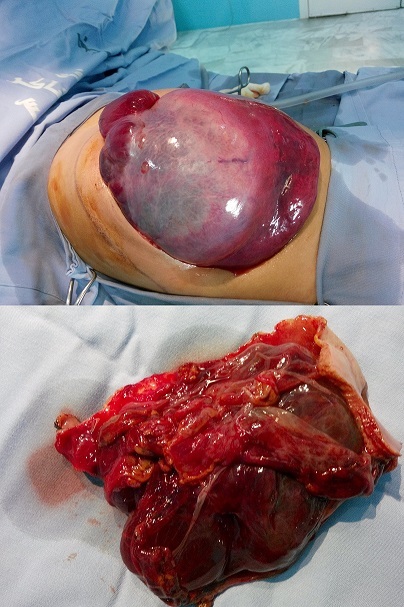
Intraoperative view and resected specimen showing the cystic lymphangioma

